# Endogenous avidin biotin activity (EABA) in thyroid pathology: immunohistochemical study

**DOI:** 10.1186/1756-6614-2-5

**Published:** 2009-04-08

**Authors:** Barbara Nikiel, Mykola Chekan, Michal Jarząb, Dariusz Lange

**Affiliations:** 1 Department of Tumor Pathology, Maria Skłodowska-Curie Memorial Center and Institute of Oncology, Gliwice Branch, Gliwice, Poland; 2 Department of Nuclear Medicine and Endocrine Oncology, Maria Skłodowska-Curie Memorial Center and Institute of Oncology, Gliwice Branch, Gliwice, Poland; 3 Department of Tumor Biology, Maria Skłodowska-Curie Memorial Center and Institute of Oncology, Gliwice Branch, Gliwice, Poland

## Abstract

**Background:**

Immunohistochemical methods based on the high affinity of avidin and biotin (e.g. ABC, LSAB) are characterized by high sensitivity and are widely used for detection of immunologic reaction. However, a non-specific reaction, observed in frozen tissues and in paraffin-embedded material, increasing after heat induced epitope retrieval (HIER), and caused either by endogenous biotin or any another chemical compound with high affinity for avidin, may lead to diagnostic mistakes. The aim of our investigation is to study presence of endogenous avidin biotin activity (EABA) in thyrocytes originating from various thyroid pathological lesions (neoplastic and non-neoplastic).

**Materials and methods:**

The immunohistochemical study was performed on paraffin-embedded specimens of surgically resected thyroid tissue from 97 patients with thyroid diseases: 65 patients with papillary carcinoma (PTC), 11 patients with nodular goiter in whom features of benign papillary hyperplasia were found, 9 with lymphocytic thyroiditis (LT), 8 with follicular adenoma, and 4 patients with follicular carcinoma. In PTC immunohistochemical study was performed both in primary tumors and in lymph node metastases. After HIER, incubation with streptavidin from LSAB+ (DakoCytomation) kit was done.

**Results:**

Strong cytoplasmic EABA was observed in 56 of 65 (87.5%) PTC and in oxyphilic cells in 8 of 9 cases of LT. Significant correlation between EABA in primary PTC tumor and EABA in lymph node metastases was stated. Normal surrounding thyroid tissues showed absence or weak EABA. Aberrant intranuclear localization of biotin was noted in morules of cribriform-morular variant of PTC. No statistically significant correlation between patient's age, sex, metastases presence and EABA was observed.

**Conclusion:**

Among thyroid lesions, false positive reactions are highly probable in papillary thyroid carcinoma and in lymphocytic thyroiditis if immunohistochemical detection is used on systems containing (strept)avidin. The most probable reason is the high endogenous biotin content.

## Background

For immunohistochemical diagnostics of thyroid diseases, it is very important to choose an optimal visualization method and an appropriate quality control. The evaluation has to include control for endogenous biotin, which is widely distributed in tissues and may cause non-specific staining by binding to avidin.

Biotin is a cofactor required by enzymes that are involved in carboxylation reactions, e.g. pyruvate carboxylase, which catalyses the conversion of pyruvate to oxaloacetate in initiation of gluconeogenesis and acetyl-CoA carboxylase that catalyzes the committed step in fatty acid biosynthesis [1]. The best-known and well understood role of biotin is to form the prosthetic group of the five biotin-containing carboxylases. Biotin is covalently bound to a lysine residue in acetyl-CoA carboxylase 1 (ACC-1), and acetyl-CoA carboxylase 2 (ACC-2), propionyl-CoA carboxylase (PCC), pyruvate carboxylase (PC) and methylcrotonyl-CoA carboxylase (MCC). This explains its obligatory involvement in the metabolism of carbohydrates, lipids and deaminated residues of some amino acids. Moreover, another biotin function as gene expression regulator was reported recently [2,3].

Immunohistochemical methods based on the high affinity of avidin or streptavidin and biotin are wide known and used routine methods (e.g. ABC, LSAB) and are characterized by high sensitivity. However, a non-specific reaction can be observed in frozen tissues and in paraffin embedded material, evoked by high temperature (during warming in microwave oven or bain-marie), caused by endogenous biotin or another chemical compound with high affinity for avidin. Endogenous avidin biotin activity (EABA) is present in a wide range of epithelial tissues, especially in glandular epithelia, both normal and tumor-derived [4]. Some of the thyroid neoplasms exhibit a lot of endogenous biotin that limits or excludes possibility of visualization by immunostaining with methods based on avidin-biotin combined with selected antigen retrieval [4-8].

In our investigation, we were interested in a more detailed evaluation of EABA presence in thyrocytes originating from various thyroid pathological lesions, mainly neoplastic, but also non-neoplastic ones. For comparison with papillary carcinoma (PTC), we choose those cases of nodular goiter, where signs of papillary hyperplasia were present. We were looking for association between presence of EABA in thyrocytes and factors related to the host (age and sex), factors intrinsic to the tumor (histological type), and others factors that reflect the relationship between the host and the tumor (local lymph node metastases).

## Materials and methods

The study was conducted on surgically resected thyroid tissue from 97 patients with thyroid diseases. There were 65 patients with PTC (classical (53), follicular (10) and oxyphilic (1) cribriform-morular (1) variants), 20 cases of PTC (15 cases classical variant and 5 cases follicular variant have lymph node metastases), 11 patients with nodular goiter and features of benign papillary hyperplasia, 9 with lymphocytic thyroiditis, 8 with follicular adenoma, 4 patients with follicular carcinoma (FTC) (Table 1). Age range was 8 – 78 and mean age 44 years. There were 81 women and 16 men among the studied patients.

**Table 1:** Patient's diagnosis, sex, middle age and presence of nodular metastases

Diagnosis	Patients sex			
			
	M	F	Patients Age (mean, SD)	Lymph Node metastases
PTC (all)	14	51		

PTC (classical variant)	10	43	46 ± 17.22	19

PTC (follicular variant)	4	6	32 ± 18.52	4

PTC (oxyphilic variant)	0	1	42	0

PTC (cribrifirm-morular variant)	0	1	22	0

Nodular goiter with benign nodular hyperplasia	1	10	44 ± 13.71	-

Lymphocytic thyroiditis	0	9	50 ± 13.07	-

Follicular adenoma	1	7	46 ± 12.84	-

FTC	0	4	48 ± 14.49	0

Paraffin-embedded specimens were used in the immunohistochemical study. Sections were cut for 5 μm and mounted on Poly-L-lysine coated slides. After deparafinisation, antigen retrieval was carried out in citrate buffer pH = 6.0 by microwave treatment (3 cycles for 5 minutes, 750 W). After cooling, the sections were immersed in 3% hydrogen peroxide to block endogenous peroxidase activity – 5 minutes, then washed in TBS buffer pH = 7,6 for 5 minutes. Incubation with streptavidin from LSAB+/HRP kit (DakoCytomation) lasted 25 min, then the slides were washed in TBS buffer 2 times for 5 minutes and incubated with DAB for 10 min, washed in distilled water and stained with hematoxylin for 1 minute. For control purposes, all cases were also evaluated by EnVision visualization system (DakoCytomation). Micrographs were done with Carl Zeiss Axioplan 2 microscope, Sony 3CCD color video camera using KS400 (Carl Zeiss) software.

## Results

**Figure 1:**
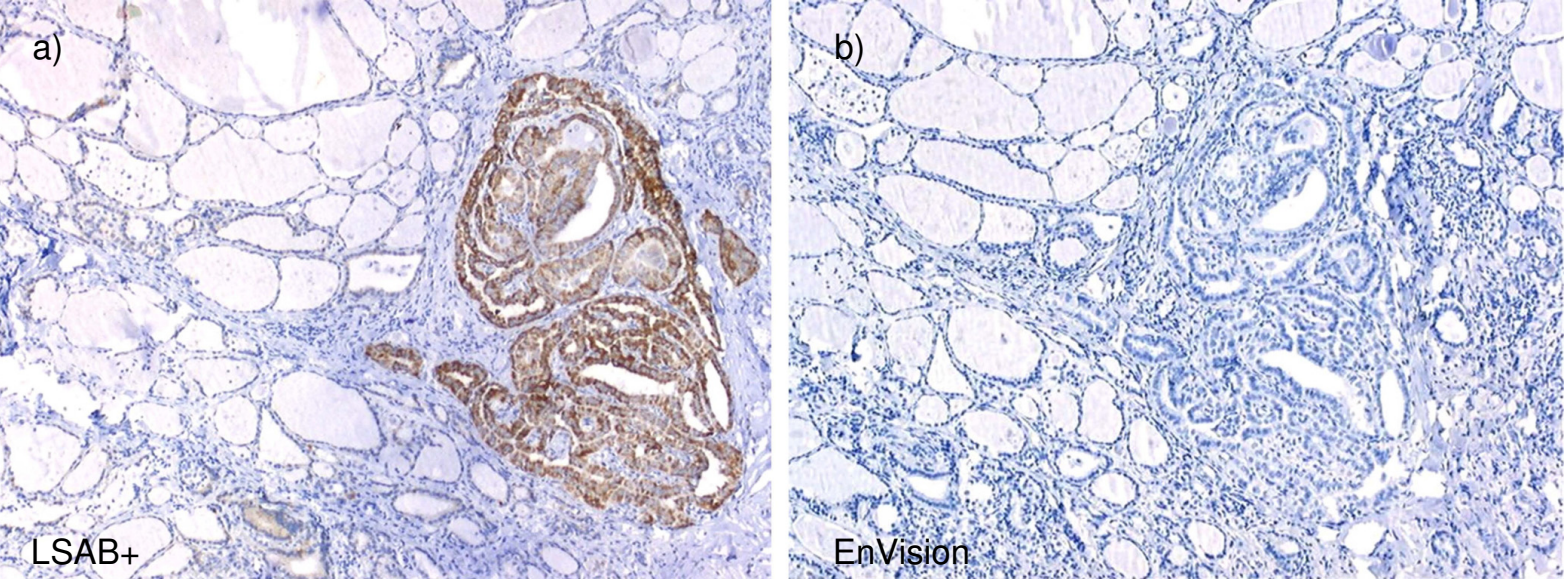
**Cytoplasmic EABA in PTC tissue**. a) Cytoplasmic EABA in papillary thyroid carcinoma cells (LSAB+); objective magnification 5×; b) No reaction in the same sample of PTC (EnVision); objective magnification 5×.

Both cytoplasmic and nuclear reaction with endogenous biotin was observed in thyroid tissue. The EABA-positive results were obtained in thyroid glands of 72 patients, among them 57 of 65 PTC cases, 1 of 11 nodular goiter cases showing signs of benign papillary hyperplasia, 8 of 9 lymphocytic thyroiditis cases, 1 of 4 FTC cases. The most intensive EABA was observed in cases of PTC and lymphocytic thyroiditis (Table 2). Essentially to note, no immunostaining was observed using EnVision visualization system that does not contain avidin or streptavidin. Consequently, nonspecific staining resulting from EABA was eliminated (Figure 1).

**Table 2:** EABA intensity in different thyroid lesions

Thyroid disease	Number of positive cases	EABA Intensity
PTC (all)	57/65	+++

PTC (classical variant)	47/53	+++

PTC (follicular variant)	8/10	++

PTC (oxyphilic variant)	1/1	+++

PTC (cribrifirm-morular variant)	1/1	+++ (nuclear)

Nodular goiter with benign nodular hyperplasia	1/9	++

Lymphocytic thyroiditis	8/9	+++

Follicular adenoma	4/7	+

Follicular carcinoma	1/4	++

Note: + brown staining in less then 20% of cells; ++ brown staining between 20% – 60% of cells; +++ brown staining more than 60% of cells.

The majority of PTC (87.7%) showed intensive EABA. The reaction was weaker in follicular variant of PTC. Positive cytoplasmic EABA in histologically normal surrounding tumor thyroid tissue was observed in 61.8% of PTC cases. In all cases of positive surrounding tissue reaction, it was weak with fewer positive cells than observed in tumor tissue. No statistically significant correlation was found between EABA and patients' sex, age, or, in the case of cancer cases, the lymph node metastases presence. A positive correlation between EABA intensity in primary tumor and EABA intensity in lymph node metastases was observed (Spearman R = 0.83 p-level = 0.008).

**Figure 2:**
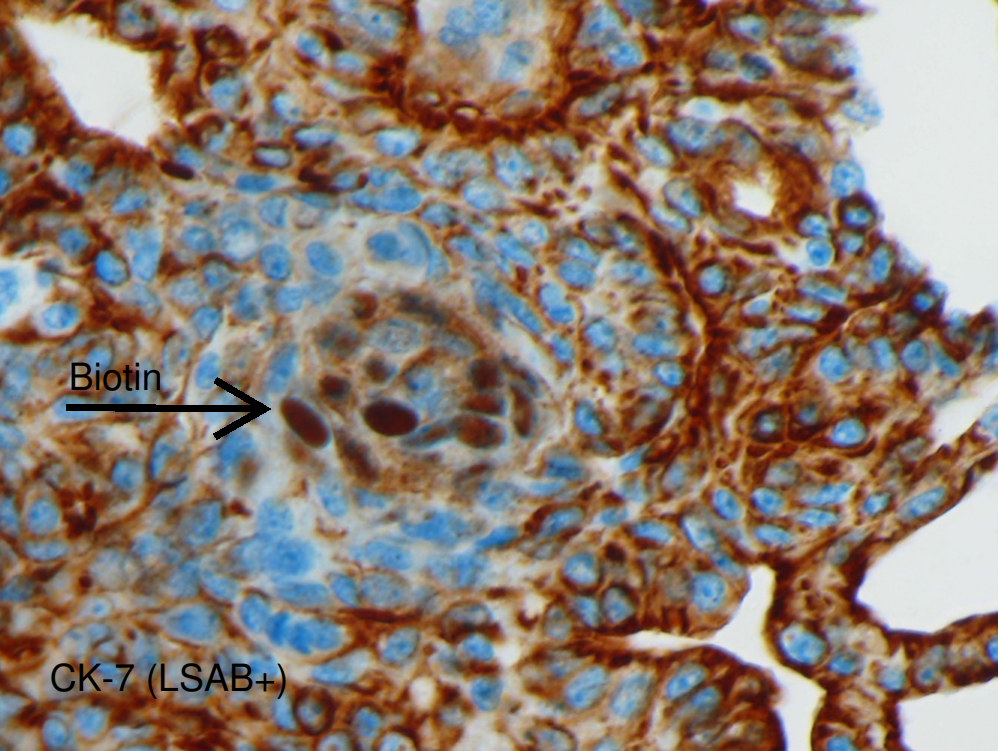
**Aberrant biotin in nuclei of morular cells of PTC cribrifirm-morular variant**. CK-7 stain (LSAB+ visualization method) glandular cells show CK-7 expression (membranous reaction); aberrant biotin present in nuclei of morular cells. Objective magnification 40×.

In addition, we observed aberrant intranuclear localization of biotin in morules of cribriform-morular variant of PTC. This rare variant of PTC was seen only in one patient, 22 years old female. The reaction was observed during immunostaining for Cytokeratin 7 (CK-7). In glandular component of the tumor there was positive membranous reaction for CK-7 and negative for nuclear biotin. Morular component of the tumor was immunonegative for CK-7 but positive for biotin in nucleus (Figure 2).

**Figure 3:**
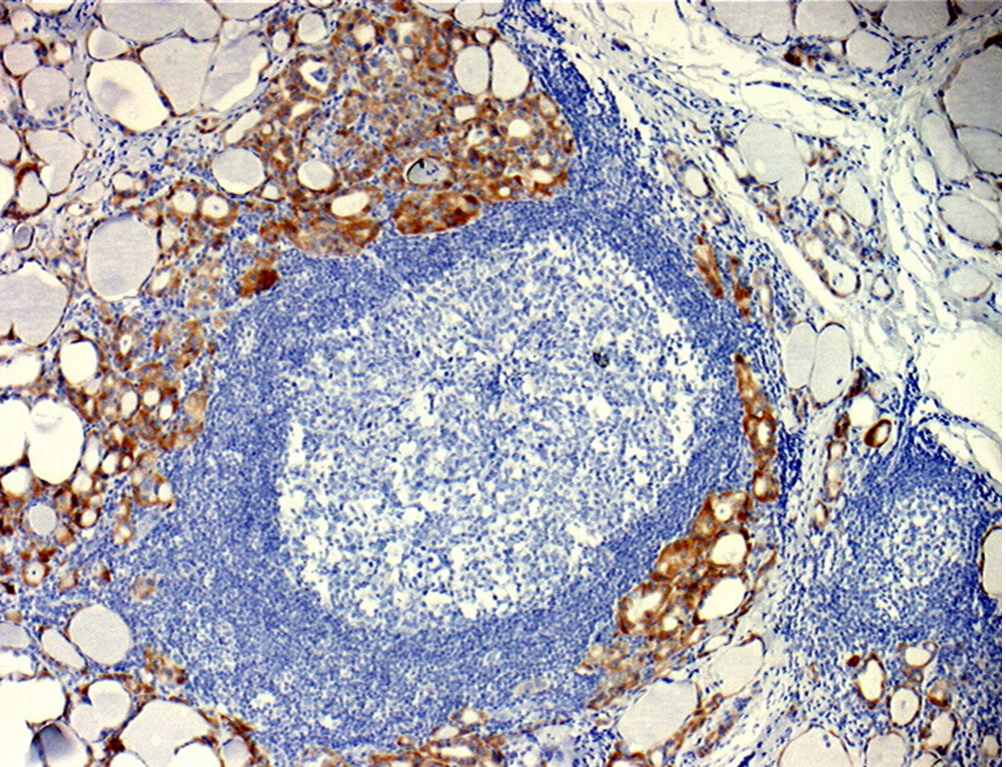
**Cytoplasmic EABA in follicular cells in lymphocytic thyroiditis**. (LSAB+); objective magnification 5×.

Cytoplasmic EABA was observed just in one case of nodular goiter and the reaction was present in oxyphilic cells. In lymphocytic thyroiditis, intensive and widespread cytoplasmic EABA was observed in thyrocytes with oxyphilic metaplasia that were located around lymphoid germinal centers (Figure 3). In follicular adenoma weak EABA was noted just in few cells. To evaluate a possible diagnostic significance of EABA in distinguishing PTC from other thyroid pathology ROC curve was calculated. The area under the curve (AUC) was 0.721.

## Discussion

Endogenous biotin activity has been indicated by previous studies dealing with thyroid immunohistochemistry (Table 3). Our study extends these data and indicates that EABA activity is present in thyroid lesions which are abundant in mitochondria (oxyphilic metaplasia, oxyphilic tumors) and in papillary thyroid cancer, both primary tumor and lymph node lesions, but not in benign goiter showing features of papillary hyperplasia. Bussolati et al. (1997) in their study proposed even constitutive endogenous biotin as a putative diagnostic marker [9]. Our study negates this possibility, as the area under ROC curve does not reach 80%.

**Table 3:** EABA in thyroid tissue according to published studies

	Antigen retrieval method	Type of thyroid lesion	Observation
Bussolati et al. (1997) [9]	Autoclaving; microwave	OTC	Expression ++ in 5/5
		
		PTC	Expression + in 3/5

Kashima K et al. (1997) [6]	Trypsin pretreatment; Autoclaving	Various thyroid lesions	Expression + in 93/208
		
		PTC and FTC	Higher expression
		
		ATC, squamous cell carcinoma; poorly differentiated carcinoma	Lowest expression

Zhou X et al. (2002) [4]	Microwaving in EGTA buffer pH 9.0; 8.0; 6.0	C cells, PTC, OTC	EABA was easier revealed by higher pH value buffer (EGTA pH 9.0) than that with lower pH value (EDTA pH 8.0 and citrate pH 6.0)

Herrmann ME et al. (2002) [13]	Microwaving in 0.1 Tris buffer pH 7.4	PTC, reactive oxyphilic cells	identified with biotin detection system when the primary antibody was omitted

Mutsomoto F et al. (2006) [12]	Microwaving in citric acid (pH 6.0) for 10 minutes at 100°C	oxyphilic cells	Elimination of nonspecific staining due to endogenous avidin-biotin activity by using En-Vision+ system HRP-labeled polymer

Endogenous biotin immunoreactivity was generally not visualized in formalin-fixed, paraffin-embedded tissues unless a HIER (heat-induced epitope retrieval) step has been introduced. The thermal denaturation of tissue sections by microwave pre-treatment or pressure-cooking techniques is used to unmask tissue epitopes exposing endogenous biotin and thus can result in spurious immunohistochemical staining [10,11].

Endogenous biotin levels in human cells are especially high in liver, renal tubular epithelium, brown fat and adrenal cortex. Bussolati et al. (1997) was the first who showed that numerous other human tissue types might demonstrate endogenous biotin expression following antigen retrieval processes (autoclaving or microwaving) and listed also PTC (3 of 5 studied) and oncocytic type of follicular thyroid carcinoma (OTC) (5 of 5 studied) among analyzed human neoplasms in this study [9]. In study of Zhou X et al. (2002) EABA in PTC and OTC was easier revealed by higher pH value buffer (EGTA pH 9.0) than that with lower pH value (EDTA pH 8.0 and citrate pH 6.0). Kashima K et al. [6] demonstrated that cytoplasmic biotin-like activity could be identified in formalin-fixed and paraffin-embedded human thyroid lesions by immunostaining for biotin using peroxidase-antiperoxidase method or by peroxidase-labeled streptavidin alone. The reactivity of cytoplasmic biotin-like activity was markedly enhanced both by pretreatment with trypsin and after heating by autoclaving. The positive incidence of cases and positive cell ratio were the highest in papillary carcinoma, followed by follicular carcinoma and the lowest in anaplastic carcinoma, squamous cell carcinoma and poorly differentiated insular carcinoma.

Also, thyroid oxyphilic cells are known to have high endogenous biotin and are known to show a false-positive immunohistochemical staining [12]. Oxyphilic cells were identified with biotin detection system when the primary antibody was omitted after microwaving in Tris buffer pH = 7.4 [13].

Among carboxylases requiring biotin as their coenzyme, PC, PCC, MCC and ACC-2 are located in mitochondria, ACC-1 is the only one known biotin-dependent carboxylase located in human cell cytoplasm. For this reason, much of the false positivity caused by endogenous biotin in immunohistochemical studies is a cytoplasmic immunoreaction which is found in tissues with abundant mitochondria [10], when avidin and streptavidin, closely related tetrameric proteins, which share a strong affinity for biotin, are used. This extremely strong non-covalent interaction is the basis for multitude of non-radioactive techniques used in the detection of macromolecules.

Human organism is unable to synthesize biotin and thus depends entirely on the vitamin presence in food to satisfy its vitamin requirements. To deal with their biotin requirements, humans have evolved to a very efficient and complex biotin cycle to ensure adequate supply and utilization of the vitamin. There are two enzymes involved in biotin metabolism: halocarboxylase synthetase (HCS), which covalently attaches biotin to carboxylases and biotinidase (BTD), the enzyme responsible for biotin recycling. BTD catalyzes the hydrolysis of biocytin, a normal product of biotin-dependent carboxylase degradation, to biotin and lysine, thus allowing biotin to be recycled and used in biotinylation of new carboxylases [14]. The biotin-binding/carrier function of BTD has been demonstrated. It can be responsible for transporting biotin into cells through a specific cell membrane receptor [15,16]. In addition, biotin-transferase activity of BTD in modification of genes expression by biotinylation of histones was shown [17-20]. Biotinidase activity is particularly high in liver, adrenal gland and kidney [15].

Previous studies demonstrated that cytoplasmic EABA was caused by biotin-containing carboxylases. Praul et.al (1998), using Western-blot, detected three biotin-containing carboxylases (PC, MCC and PCC) utilizing streptavidin alone [21] and concluded that EABA was higher in tissues with high rate of metabolism. On the other hand, the cause and mechanism of biotin storing is still not exactly clear [15,16,22].

Although the main biotin rich fractions are the cytosol and the mitochondria, there is some controversy as to whether moderate or negligible amounts of biotin are present in the nuclei of cells as well. Biotin was discussed to be present in optically clear nuclei of ovarian endometrioid carcinoma, pancreatoblastoma, pulmonary blastoma, pulmonary endodermal tumor and morules within colonic tubular adenoma and thyroid carcinoma [10]. It was suggested that the observed phenomena were caused by biotinylation of histones or might be indirect through the involvement of biotin in the synthesis of ATP and protein enzymes [10,15,16]. In our study, we also observed nuclear EABA in cribriform-morular variant of PTC nuclei.

Majority of authors consider EABA in thyroid tissue to be an artifact that limits or even excludes using avidin/streptavidin-containing detection systems for immunochemistry. It is important to note that immunohistochemical profile of PTC cells is very similar to that exhibited by the follicular cells in lymphocytic thyroiditis, a fact that should be taken into account in the differential diagnosis between the two entities and before the making a claim about the "specificity" of a given antibody to PTC [23].

In addition to the diagnostic and biological aspects, the present study is obviously of methodological interest in a wider sense. Although not every application of the (strept)avidin/biotin technology is necessarily affected by endogenous biotin, the problem certainly extends beyond immunohistochemistry (e.g. the recent proposals to introduce in vivo therapies based on application of biotin). Therefore, precautions should be taken by determining whether endogenous biotin interferes with avidin-biotin applications.

## Conclusion

Endogenous avidin biotin activity presence in papillary thyroid carcinoma cells and oxyphilic cells can cause cytoplasmic and nuclear false-positive immunohistochemical staining. Thus, using of visualization systems that do not contain (strept)avidin is necessary for immunohistochemistry of thyroid disease. If it is necessary to use streptavidin-containing detective systems, the examination of tissue-specific negative controls with every immunohistochemical study is necessary. However, we do not support use of EABA for distinguishing PTC from other thyroid lesions because of its frequent presence in thyroid oxyphilic cells.

## Competing interests

The authors declare that they have no competing interests.

## Authors' contributions

NB conceived the study, carried out the immunohistochemical staining, participated in scoring of immunohistochemical reaction and participated in the preparation of the manuscript. CM performed scoring of immunohistochemical reaction, performed the statistical analysis and drafted the manuscript. JM participated in the design of the study and statistical analysis. LD confirmed the diagnosis of every studied sample and participated in study design and coordination. All authors read and approved the final manuscript.
